# Design and Characterization of Electrochemical Sensor for the Determination of Mercury(II) Ion in Real Samples Based upon a New Schiff Base Derivative as an Ionophore

**DOI:** 10.3390/s21093020

**Published:** 2021-04-25

**Authors:** Salman S. Alharthi, Ahmed M. Fallatah, Hamed M. Al-Saidi

**Affiliations:** 1 Department of Chemistry, College of Science, Taif University, P.O. Box 11099, Taif 21944, Saudi Arabia; a.fallatah@tu.edu.sa; 2 Department of Chemistry, University College in Al–Jamoum, Umm Al–Qura University, Makkah 21955, Saudi Arabia; hmsaidi@uqu.edu.sa

**Keywords:** Schiff base, mercury selective electrode, ionophore, PVC membrane, ISE-Hg

## Abstract

The present paper provides a description of the design, characterization, and use of a Hg^2+^ selective electrode (Hg^2+^–SE) for the determination of Hg^2+^ at ultra-traces levels in a variety of real samples. The ionophore in the proposed electrode is a new Schiff base, namely 4-bromo-2-[(4-methoxyphenylimino)methyl]phenol (BMPMP). All factors affecting electrode response including polymeric membrane composition, concentration of internal solution, pH sample solution, and response time were optimized. The optimum response of our electrode was obtained with the following polymeric membrane composition (% *w*/*w*): PVC, 32; *o*-NPOE, 64.5; BMPMP, 2 and NaTPB, 1.5. The potentiometric response of Hg^2+^–SE towards Hg^2+^ ion was linear in the wide range of concentrations (9.33 × 10^–8^−3.98 × 10^–3^ molL^–1^), while, the limit of detection of the proposed electrode was 3.98 × 10^–8^ molL^–1^ (8.00 μg L^–1^). The Hg^2+^–SE responds quickly to Hg^2+^ ions as the response time of less than 10 s. On the other hand, the slope value obtained for the developed electrode was 29.74 ± 0.1 mV/decade in the pH range of 2.0−9.0 in good agreement with the Nernstian response (29.50 mV/decade). The Hg^2+^–SE has relatively less interference with other metal ions. The Hg^2+^–SE was used as an indicator electrode in potentiometric titrations to estimate Hg^2+^ ions in waters, compact fluorescent lamp, and dental amalgam alloy and the accuracy of the developed electrode was compared with ICP–OES measurement values. Moreover, the new Schiff base (BMPMP) was synthesized and characterized using ATR–FTIR, elemental analysis, ^1^H NMR, and ^13^C NMR. The PVC membranes containing BMPMP as an ionophore unloaded and loaded with Hg(II) are reported by scanning electron microscope images (SEM) along with energy-dispersive X-ray spectroscopy (EDX) spectra.

## 1. Introduction

Mercury exists in nature at trace and ultra-trace levels. However, it is one of the most toxic heavy metals on earth. Among the different valence states, Hg (II) is the most toxic even when present in very trace amounts [1]. Mercury can enter and accumulate in the human body through the food chain causing severe health problems such as vital organ damage, nervous system impairment, kidney failure, and cancer [2,3,4,5]. Thus, monitoring trace concentrations of this toxic element has become a vital necessity. Various analytical techniques have been developed and used for the determination of mercury species in different samples. These techniques include spectroscopic measurements in the UV-Vis region [6], cold vapor atomic absorption spectrometry (CV–AAS) [7], atomic emission spectroscopy (AES) [8], cold vapor atomic fluorescence spectrometry (CV-AFS) [9], measurements using inductively coupled plasma mass spectrometry (ICP–MS) [10], X-ray fluorescence [11], ion chromatography [12,13] and electrochemical sensors [14]. Despite the fact that these techniques have high sensitivity and accuracy, they have some disadvantages in terms of high costs, maintenance, and complicated data analysis. Furthermore, highly trained skilled technicians are needed for data interpretation and operation [15]. As a result, developing a simple, fast, low-cost, accurate, sensitive, and selective analytical technique is necessary. Since it is relatively inexpensive, simple to operate, and provides a real-time measurement, the ion-selective electrode (ISE) is one of the most popular electroanalytical techniques used to determine the concentration of a wide variety of metal ions in various samples such as food, soil, and waters. Therefore, ISE can monitor the change of activity of ion with time [16,17,18].

Many organic and inorganic compounds have been documented in the literature as ionophores for the synthesis of ion selective electrodes for the determination of Hg^2+^ ion in various samples over the last three decades. Crown ether derivatives [19,20], polyvinyl pyridine [21], calix[4]pyrrole amide derivative [22], calixarene derivatives [23,24,25,26,27,28], thiol functionalized ionic liquid [29], amines [30,31], thioureas [25,32], and dithio derivatives [33,34] have all been used as ionophores for designing ISEs of Hg^2+^ ions. It’s worth mentioning that, some ionic liquids, e.g., 1-n-butyl-3-methylimidazolium tetrafluoroborate (BMIMBF_4_) [35], polypyrrole (PPy) [36], O,O′-(2,2′-biphenylene)dithiophosphatepentyl (PenDTF) [37], 1-methyl-2-butylthioimidazolium combined with bis(trifluoromethanesulphonyl) imide [29], dimethylglyoxime [38], and 2-mercaptobenzimidazol (2MBI) [39] are widely employed as ionophores in ISEs of Hg(II) ions.

It is well known that Schiff bases can form stable complexes with most transition metal ions including mercury (II) ions [40,41]. Thus, many of these bases were prepared and used as ionophores for developing ISEs of mercury (II) [42,43,44,45,46]. Due to the good sensitivity of the selective electrodes dependent on Schiff bases, the present work will aim to prepare a new Schiff base and its use as a neutral ionophore for construction of Hg^2+^ selective electrode. Electrode membrane containing a new Schiff base will be characterized before and after loading Hg^2+^ ions using SEM micrographs and EDX spectra. The developed electrode will use for the determination of mercury in some real samples.

## 2. Experimental

### 2.1. Reagents and Chemicals

All reagents and chemicals used in this work were of analytical grade and used without further purification. Bromo-2-hydroxybenzaldehyde, 4-methoxyphenyl amine, *o*-nitro-phenyloctylether, sodium tetraphenylborate, high molecular weight polyvinyl chloride (PVC), and metal salts (as nitrates) were purchased from MilliporeSigma (Saint Louis, MO, USA). and used with no further purification. Organic solvents were obtained from Thermo Fisher Scientific (Hampton, NH, USA). All solutions were prepared using deionized water.

### 2.2. Instrumentation

A Perkin Elmer analyzer model 2400 (Waltham, MA, USA) was used for determining the elemental compositions of the BMPMP ligand. A ^1^H NMR and ^13^C NMR spectrum of the BMPMP ionophore was obtained in DMSO-d_6_ solvent using Bruker FT-NMR spectrometer (model DRX-500, Billerica, MA, USA). ATR-FTIR spectra of membrane used in Hg^2+^–SE was recorded in the range of 400–4000 cm^–1^ using JASCO 4600 FT-IR spectrometer (Tokyo, Japan). Samples were directly introduced using the unit of the attenuated total reflectance model ATR PRO ONE Single-Reflection. Morphological studies and elemental distributions on the synthesized electrode surface were investigated using a JEOL JEM-6390 scanning electron microscope combined with a unit of energy-dispersive X-ray spectroscopy (Peabody, MA, USA). Determination of concentration of mercury in aqueous solutions was carried out by Perkin Elmer ICP–OES spectrometry model Optima 2100 DV (Waltham, MA, USA). Acidic reaction (pH) measurements were performed using an advanced bench pH meter (model 3510, Jenway, Staffordshire, UK). pH–meter was calibrated by three different buffer solutions (pH 4.01-AD7004, pH 7.01-AD7007, and pH 10.01-AD7010). Potentiometric measurements (EMF, mV) of the designed Hg^2+^–SE were recorded under different conditions using a silver/silver chloride with Flexible Connector (MF-2052, RE-5B) filled with sodium chloride solution (3 molL^–1^) as an external reference electrode. The electrochemical cell used for carrying out potentiometric measurements consists of Ag(s), AgCl(s)/internal solution (1.0 × 10^−2^ molL^−1^ Hg^2+^ ion in 0.1 molL^–1^ KCl)/ISE membrane/sample solution/pH glass electrode.

### 2.3. Synthesis of (4-Bromo-2-[(4-Methoxyphenyl-Imino)Methyl]Phenol, BMPMP

**Scheme 1:**
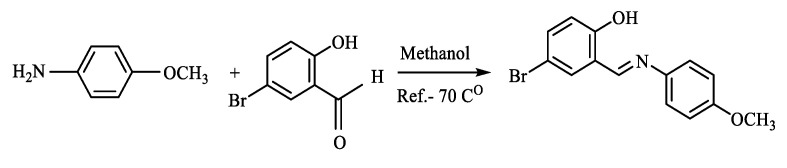
Synthetic protocol of (4-bromo-2-[(4-methoxyphenylimino)methyl]phenol, BMPMP.

4-Bromo-2-[(4-methoxyphenylimino)methyl]phenol was synthesized according to the methodology reported in the literature [47]. A mixture of 4-methoxyphenyl amine (25 mmol) and 5-bromo-2-hydroxybenzaldehyde (25 mmol) was refluxed in methanol (50 mL) for 3 h at 70 °C. The precipitate was filtered, washed, and recrystallized by a mixture of methanol and diethyl ether. Yield, 0.259 g (71.7%), m.p.: 150 °C. MS: *m*/*z* 306.58 [M]^+^. Elemental analysis was carried out in duplicates; (CHN) for C_14_H_12_O_2_NBr (MW. 306.16): Calculated (C%, 54.92; H%, 3.95; N%, 4.57). Found (C%, 54.65; H%, 3.76; N%, 4.48). ^1^H NMR (500 MHz, DMSO-d_6_): δ = 2.60 (s, 3H, CH_3_), 7.55–7.98 (m, 7H, aromatic H), 9.05 (s, 1H, CH = N), 14.22 (s, 1H, OH). ^13^C-NMR (DMSO-d_6_, 500 Mz): δ = 67.00 (COCH_3_), 109.6 (Br-C), 112.4 (CHCHCOCH_3_), 119.1 (CHCHCOH), 121.6 (CHCCNC), 124.6 (NCCH), 129.7 (BrCHC), 133.7 (BrCHCH), 140.5 (NCCH), 157.1 (CHCOH), 159.8 (COCH_3_), 163.9 (CC = N) [supplementary file]. Scheme 1 shows the protocol of BMPMP synthesis. 

### 2.4. Construction of Membrane Electrode

Nine membranes were prepared using the technique described in the literature, with different concentrations of polymer (PVC), ionophore (BMPMP), ionic additive (NaTPB), and plasticizer (o-NPOE). [42]. A mixture of previous components with various percentages shown in Table 1 was dissolved in 6 mL tetrahydrofuran (THF) with shaking for 5 min. The solution was transferred into a petri dish and left at room temperature (25 ± 2 °C) until solvent was evaporated. Thereafter, a tube with a diameter of 15 mm was immersed into the previous mixture for about 10 s to obtain a transparent membrane with the aid of an adhesive solution prepared by dissolving PVC in THF. After 24 h, the tube was separated from the mixture and filled with an internal solution of saturated KCl containing Hg(NO_3_)_2_ (1 × 10^–3^ molL^–1^). The internal reference electrode was Ag/AgCl electrode. The membranes were conditioned overnight in a solution of Hg(NO_3_)_2_ with a concentration of 110^–2^ molL^–1^.

### 2.5. Potentiometric Measurements

Potentiometric measurements were performed on the engineered PVC membranes, with the prepared Hg^2+^ selective electrode and reference electrode inserted in 50 mL of Hg(NO_3_)_2_ solution at concentration levels of 1.00 × 10^–2^ to 1.00 × 10^–8^ molL^–1^ before the potential reading became stable. All measurements were carried out at 25 ± 2 °C and pH 6 with magnetic stirring. The potential of the electrochemical cell, including Hg^2+^–SE was calculated using the Nernst equation:

(1) $$E_{cell}=E^{o}-2.303\;\frac{RT}{zF}loga$$

where *E_cell_*, *E*°, R, *T*, and *F* are the potential of electrochemical cell potential, the standard potential, gas constant, absolute temperature, and Faraday constant, respectively, while, *z* is the ion charge, and a is its activity. The ion activities were calculated using Debye–Huckel equation [48]. Calibration curves of tested Hg^2+^–SEs were obtained by plotting *E_cell_* in mV versus—log$a_{{\mathrm{Hg}}^{2+}}$.

### 2.6. Selectivity Measurements 

The potentiometric selectivity coefficients ($K_{Hg,M}^{pot}$) of the proposed Hg^2+^–SE against interfering ions were determined according to the separate solution method (SSM) [49]. It was conducted as follows: pH value of a solution of primary ion of Hg(NO_3_)_2_ (2.23 × 10^−4^ molL^−1^) was adjusted to an optimal value of 6.0 using HCl 1 molL^−1^ and/or NaOH 1 molL^−1^. A constant concentration of the interfering ion solution (2.23 × 10^−4^ molL^−1^) was added to a solution of primary ion of Hg(NO_3_)_2_ (2.23 × 10^−4^ molL^−1^) until the same potential change (Δ*E*) was achieved. For each interferent the selectivity factor $K_{Hg,M}^{pot}$ was calculated using the following equation:

(2) $$logk_{\mathrm{Hg},M}^{pot}=\frac{E_{\mathrm{Hg}}-E_{M}}{2.303RT/Z_{A}F}+loga_{\mathrm{Hg}}-loga_{M}^{1/Z_{M}}$$

where *E_M_* is the standard potential of the interfering ion at the activity *a*_M_ and *E*_Hg_ is the standard potential of the primary ion at the activity *a*_M_.

### 2.7. Potentiometric Titration

The PVC membrane achieved the optimized response was used for designing Hg^2+^ selective electrode (Hg^2+^–SE). The designed electrode (Hg^2+^–SE) was evaluated as an indicator electrode by the potentiometric titration of 60 mL of Hg(NO_3_)_2_ solution (2.00 × 10^–3^ molL^–1^) with standard EDTA solution (3.00 × 10^–2^ molL^–1^). Calibration curve was employed to determine Hg^2+^ ion concentration accurately. 

### 2.8. Preparation of Real Samples

Four real samples of tap water, sea water, compact fluorescent lamp, and dental amalgam alloy were used to evaluate the efficiency of Hg^2+^–SE as an indicator electrode for the potentiometric determination of Hg^2+^ ions.

#### 2.8.1. Preparation of Water Samples

Tap water was sampled from laboratories of the department of chemistry, Taif University, KSA. Sea water was collected from the Red Sea, Jeddah City, western Saudi Arabia. Water samples were filtered through Whatman filter paper (No. 1 with a diameter of 150 mm). Each water sample was transferred into a clean 50-mL volumetric flask. The pH of samples was adjusted at 6 using a mixture of HCl and NaOH. The determination of Hg^2+^ ions in water samples was carried out at 25 ± 0.2 °C by potentiometric titration using the developed Hg^2+^–SE as an indicator electrode. For comparison, ICP-OES measurements were performed according to the method mentioned in [50].

#### 2.8.2. Preparation of Compact Fluorescent Lamp

A compact fluorescent lamp sample was obtained from Alfanar Company, Riyadh city, KSA. The obtained sample was treated according to the mentioned procedure [51]. Briefly, the sample was digested using a mixture of concentrated nitric acid and H_2_O_2_ with a concentration of 30% for 1 h. The solution obtained after digestion was neutralized by NaOH (5 molL^–1^) and diluted to 50 mL. A part of solution was subjected to potentiometric titration using Hg^2+^–SE as an indicator electrode for the determination of mercury in a compact fluorescent lamp.

#### 2.8.3. Preparation of a Dental Amalgam Alloy

A dental amalgam capsule alloy was purchased from Dentsply Sirona Company. According to the manufacturer, the alloy contains 33.0% Ag, 8.5% Sn, 16.5% Cu, and 42.0% Hg. An accurate weight of alloy was digested using HNO_3_ (20 mL, 60%) at 60–70 °C for 2 h. The residue was washed with deionized water and filtered into a 50-mL volumetric flask. The solution pH was adjusted at 6 using a mixture of HCl and NaOH solutions. The content of mercury in alloy was analyzed by standard addition method where change in voltage is monitored after each addition of the standard solution of Hg(NO_3_)_2_ (3.0 × 10^–3^ molL^–1^). Moreover, the mercury concentration in the dental amalgam sample was also determined using the ICP-OES method [6,45].

## 3. Results and Discussion

### 3.1. Optimization of PVC Membrane Compositions

BMPMP ligand, synthesized in this study, contains active sides (imine and phenolic OH) that may react with Hg (II) ions to form a stable complex. Therefore, this Schiff base was used as a new ionophore to prepare the selective electrode for Hg^2+^ ions. It was previously known that the potential of ion-selective electrodes (ISEs) is fundamentally dependent on the amount and nature of the ionophore, plasticizer, and lipophlic additives [52]. On the other hand, the plasticizer/PVC ratio plays a main role to obtain optimized response [53]. *o*-NPOE was chosen as plasticizer due to good solubility of membrane components as well as moderate dielectric constant while NaTPB was used as a lipophlic additive owing to its important role in increasing the sensitivity and selectivity of the electrode as well reduces anionic interference.

**Figure 1:**
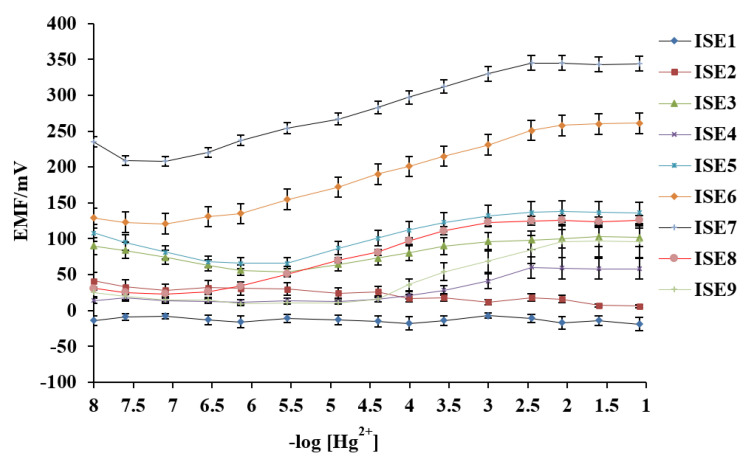
The influence of membrane composition on the potential response of the Hg^2+^-ISEs.

**Figure 2:**
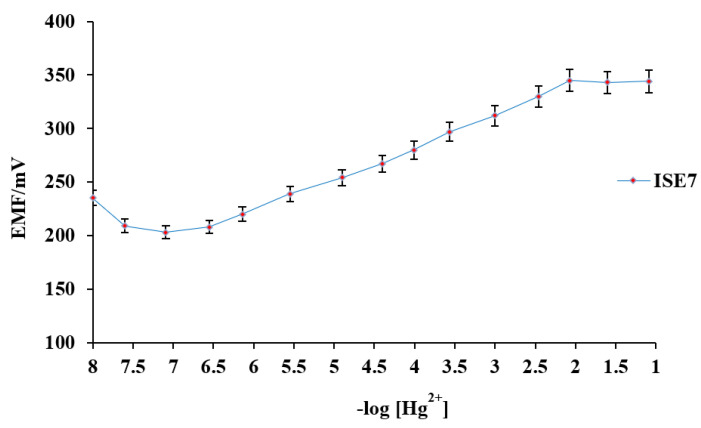
Calibration curve of the Hg^2+^-SE (ISE7) based on the BMPMP as ionophore.

Thus, the impact of membrane composition on the performance of the proposed Hg^2+^–SE was investigated by designing nine ISEs containing different membranes as shown in Table 1. Calibration curves were plotted for each ISE and shown in Figure 1. Findings in Table 1 and Figure 1 reveal that the electrodes of ISE1 and ISE2 did not respond to the change in Hg^2+^ concentration. This behavior is most likely attributed to the absence of the BMPMP ionophore in the membrane matrix. However, membranes grafted with BMPMP as an ionophore provided better responses towards Hg^2+^ ion (ISEs 3–9). The response of these ISEs for Hg^2+^ ion may be attributed to interaction between Hg^2+^ ions and BMPMP molecule. ISE7 provided the optimized response where Nernstian slope was 29.78 ± 0.15 mV/decade in good agreement with the value of 29.5 mV/decade of the divalent ions. Moreover, ISE7 gives fast and linear response over a wide range of Hg^2+^ ion concentration (9.33 × 10^–8^–3.98 × 10^–3^ molL^–1^) with the detection limit of 3.98 × 10^–8^ molL^–1^ at optimized experimental conditions (Figure 2). Thus, PVC membrane of ISE7 was studied using ATR–FTIR, SEM micrographs, and EDX spectra before and after soaking in an aqueous solution of Hg^2+^ ions. The electrodes of ISE3, ISE4, ISE5, ISE6, ISE8, and ISE9 provided low performance compared with that of ISE7. This behavior is most likely attributed to saturation of membrane and its inhomogeneity [54]. The composition of ISE7 was selected for designing an ion-selective electrode for mercury determination in a variety of real samples. 

### 3.2. ATR–FTIR Investigation of Hg^2+^–SE Membrane Based on BMPMP as an Ionophore

**Figure 3:**
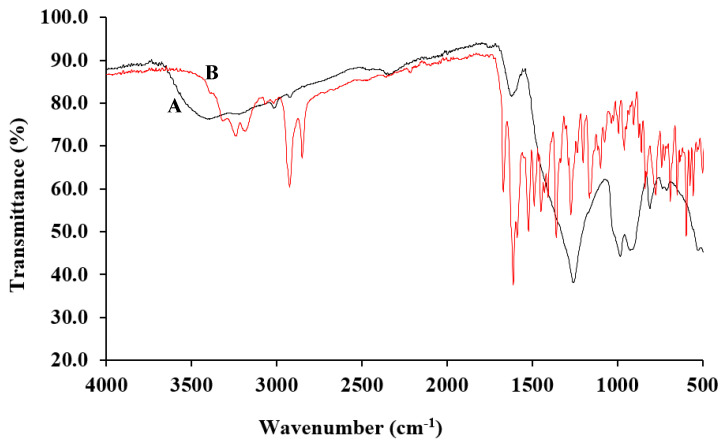
ATR–FTIR spectra of Hg^2+^–SE membrane: (A) membrane before being superimposed in Hg^2+^–SE and (B) the membrane after using for sensing Hg^2+^ ions.

ATR–FTIR spectra of Hg^2+^–SE membrane containing optimized composition were recorded before and after using for sensing Hg^2+^ ions to obtain information on the ion–ligand interaction and specify the active sites available in BMPMP molecule that can coordinate with Hg^2+^ ion. A thin layer of membrane was used to record ATR–FTIR spectra shown in Figure 3. ATR–FTIR spectrum of PVC membrane that does not contain BMPMP was recorded and subtracted from the spectra shown in Figure 3A,B. Characteristics bands of PVC membrane before soaking with analyte solution (Figure 3A) are observed at 3400, 1642, 1523, and 1407 cm^−1^ corresponding to ν(O–H), ν(C = N), ν(C = C), and δ(O-CCH_3_), respectively. Significant changes in the spectrum of membrane loaded with Hg(II) indicate an interaction between BMPMP and Hg^2+^ ions in a PVC membrane matrix (Figure 3B). The disappearance of peak at 3400 cm^−1^ corresponding to ν(OH) and the appearance of a new peak of ν(Hg–O) at 549 cm^−1^ reveal that BMPMP ligand has coordinated with Hg^2+^ ions by the phenolic OH. Coordination of Hg^2+^ ions through nitrogen atom of BMPMP molecule is confirmed by the red shift of the ν(C = N) band from 1642 to 1600 cm^−1^ and the appearance of new peak at 510 cm^−1^ corresponding to ν(Hg–N) [55]. 

### 3.3. SEM-EDX Investigations of Hg^2+^–SE Membrane

**Figure 4:**
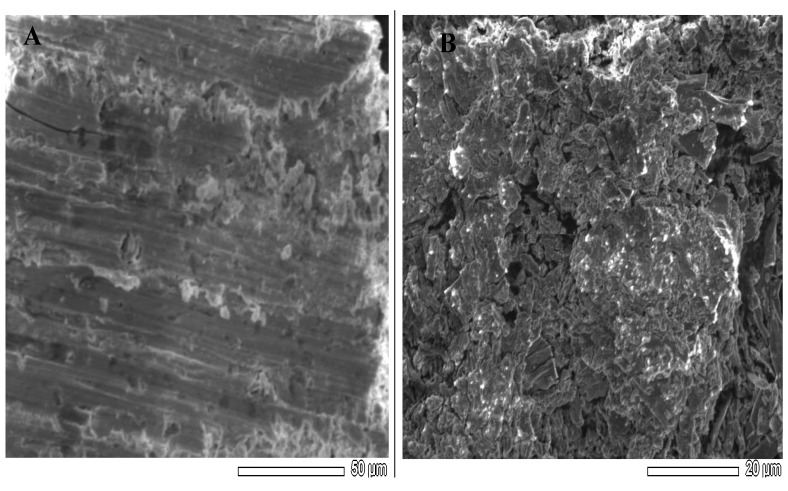
SEM micrographs of (**A**) control membrane and (**B**) optimized membrane used for sensing mercury (II).

**Figure 5:**
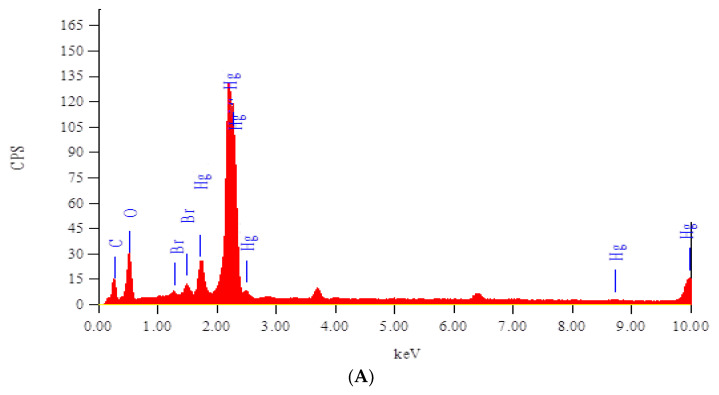

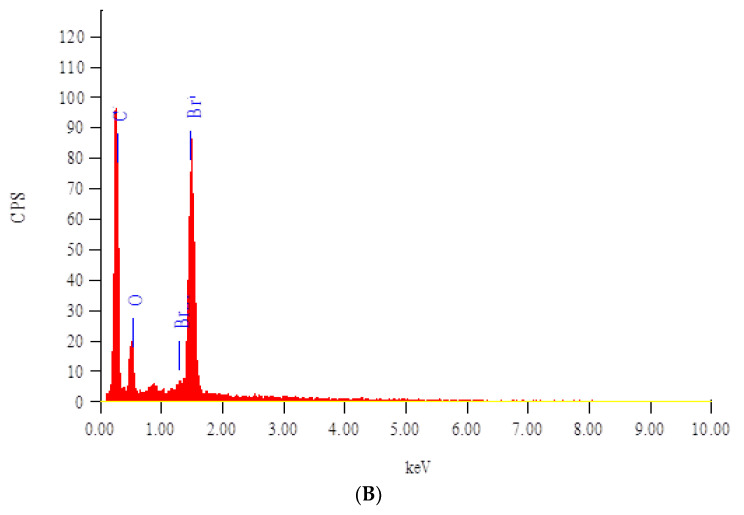
EDX spectra of (**A**) control membrane and (**B**) membrane selected for sensing mercury (II).

The morphology of the PVC membrane containing BMPMP as the ionophore was studied by SEM images before being superimposed in the ISE. The SEM micrograph shows a microporous membrane (Figure 4A). The surface of the membrane is somewhat smooth with few small protrusions. There are significant changes in membrane morphology after soaking in an aqueous Hg^2+^ solution as demonstrated in Figure 4B. The surface of the electrode is rougher with white patches spreading over the surface of the membrane providing an indication of the presence of analyte in the membrane matrix. The presence of Hg^2+^ cation in the membrane used for sensing Hg^2+^ was confirmed by EDX analysis of SEM micrograph displayed in Figure 4B. The characteristic peaks of mercury at 1.7, 2.3, and 10.2 keV were observed in Figure 5A. EDX spectrum of control membrane was recorded and shown in Figure 5B for comparison. It should be noted that the absence of the Cl peak may be due to the leaching of anionic impurities [56,57].

### 3.4. The Influence of the Internal Solution Concentration

**Figure 6:**
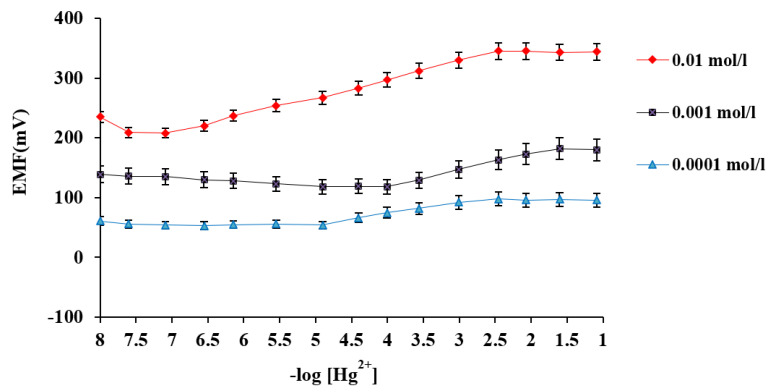
The relation between −log [Hg^2+^] and the potential of Hg^2+^–SE at different concentrations of internal solution.

An aqueous solution of Hg(NO_3_)_2_ was employed as an internal solution in the developed Hg^2+^–SE. Therefore, the concentration influence of this solution on the potential of Hg^2+^–SE was studied in the range of 1.0 × 10^–2^–1.0 × 10^–4^ molL^–1^. The results outlined in Table 2 (Figure 6) reveal that the optimized slope, wide linear concentration range, lower detection limit, and fast response time were obtained with the concentration of 1.0 × 10^–2^ molL^–1^. This is likely due to the high activity of Hg(NO_3_)_2_ solution at this concentration that enhanced the potential of Hg^2+^–SE. Thus, this concentration was employed in subsequent work. 

### 3.5. The pH Effect on the Proposed Electrode Response

**Figure 7:**
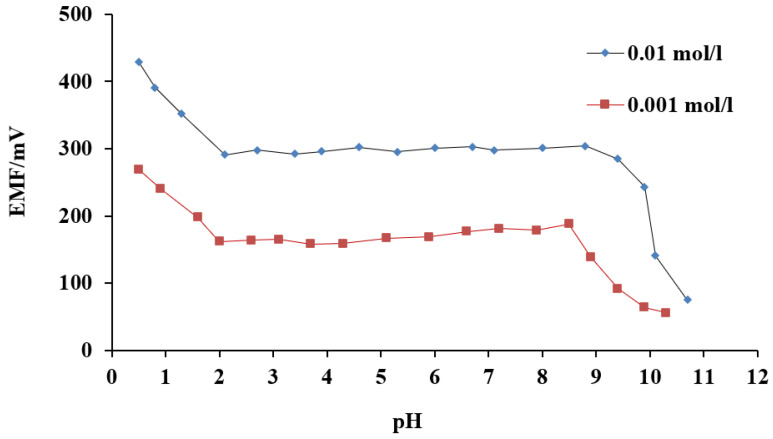
Role of test solution’s pH on the potential response of Hg^2+^–SE at different concentrations: (blue) 1.0 × 10^–2^ and (red) 1.0 × 10^–3^ molL^–1^.

Two standard solutions containing 1.0 × 10^–2^ and 1.0 × 10^–3^ molL^–1^ of Hg^2+^ ion were used to test the pH effect. The test solutions pH was adjusted to desired values (0.5–10.0) by adding HCl or NaOH (0.1 molL^–1^). Linear deficiency in the potential response was noticeable in pH range of 0.5 to 2 (Figure 7). However, the potential remains constant from pH 2.0 to 8.5. Then, a sharp deficiency was observed at higher pH values higher than 9. (Figure 7). The precipitation of Hg^2+^ ions as Hg(OH)_2_ at pH higher than 9 is a possible cause of this deficiency [58,59]. pH 6.0 was selected as the optimized value to adjust sample pH in the next work due to the fast response at this value. 

### 3.6. Response Time of the Proposed Hg^2+^–SE

**Figure 8:**
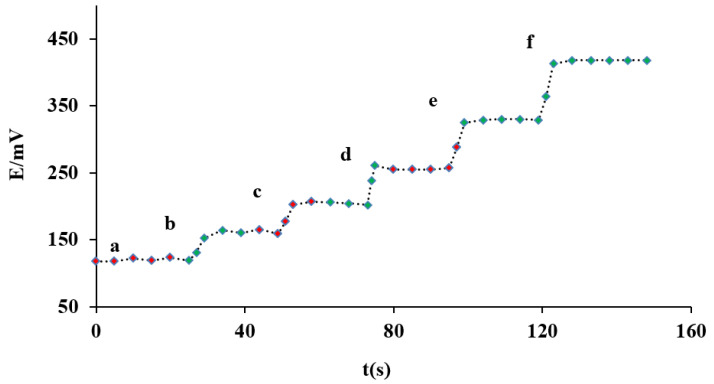
The relation of response time with Hg^2+^–SE potential at different concentrations of analyte and 298K: The curves of a, b, c, d, e, and f shows this relation at the Hg^2+^ concentrations of 1.06 × 10*^–^*^7^, 1.06 × 10*^–^*^6^, 1.06 × 10*^–^*^5^, 1.0 × 10*^–^*^4^, 1.0 × 10*^–^*^3^ and 1.0 × 10*^–^*^2^ molL^–1^, respectively.

The response time of the proposed Hg^2+^–SE was investigated at a different concentration of Hg(NO_3_)_2_ (1.0 × 10^−7^ –1.0 × 10^−4^ molL^–1^). The potential versus response time was plotted in Figure 8. The response time of developed Hg^2+^–SE becomes fast upon the concentration increases. However, the response of the electrode reached a steady-state potential in less than 10 s after analyte addition. The steadiness attained in a short response time indicates fast kinetics of Hg^2+^ ions interaction with the ionophore (BMPMP) occurring at the test solution-membrane interphase to reach chemical equilibrium [60].

### 3.7. Life Time of the Proposed Hg^2+^–SE

**Figure 9:**
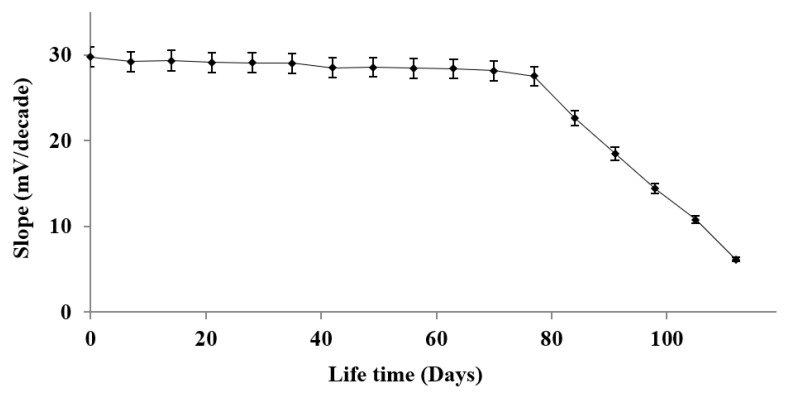
Life time of the proposed Hg^2+^-SE for a period of 112 days.

Generally, the lifetime of ISE basically relies on the electrode compositions and the number of times of use [61]. The lifetime of our electrode was investigated by measuring the slope value weekly over a 16-week period (112 days). The results shown in Figure 9 reveal that there is no significant change in the slop value (29.78 mV/decade) during the first 10 weeks. Therefore, the developed Hg^2+^–SE can be used successfully during this period for determination of Hg^2+^ ions. However, the slope value of Hg^2+^–SE decreased dramatically from 22.85 after the twelfth week to 6.15 mV/decade at 16 weeks. The expected reason for the decrease in the value of the electrode slope over time is leaching plasticizer, ionophore, additive, or PVC as a matrix from the membrane into the sample solution during use [62]. 

### 3.8. Response of the Proposed Hg^2+^–SE towards Other Ions (Selectivity)

**Figure 10:**
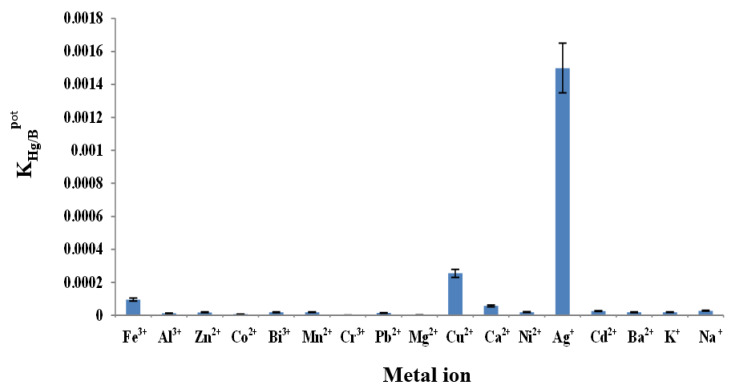
The values of $K_{Hg,M}^{pot}$ of proposed Hg^2+^–SE for variety of different cations.

The selectivity coefficient $\left(K_{A,M}^{pot}\right)$ is used to describe the influence of interfering ions on the response of ISEs. When $K_{A,M}^{pot}$ is less than 1, ISE preferentially responds to primary ion (analyte). The selectivity coefficient of Hg(II)–SE $\left(K_{Hg,M}^{pot}\right)$ was calculated according to IUPAC recommendations using the matched potential method. From findings in Table 3 and Figure 10, all tested metal ions have selectivity coefficient less than 3.0 × 10*^−^*^3^ molL^–1^. Therefore, our electrode provides high selective to the Hg^2+^ ion in the presence of wide variety of cations. 

### 3.9. Potentiometric Titrations Using Hg(II)-ISEs Based on BMPMP

**Figure 11:**
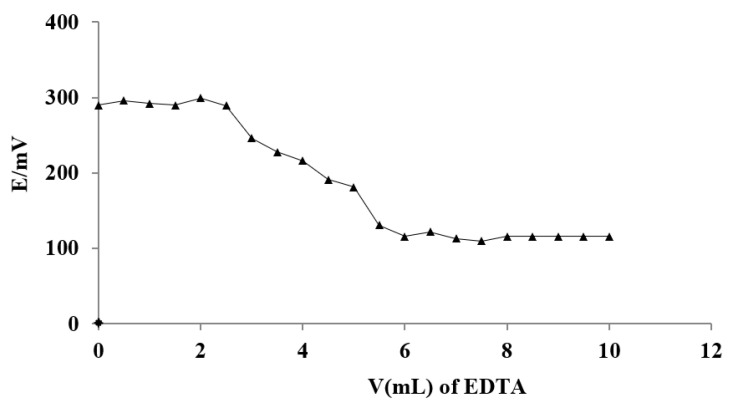
Potentiometric titration curve of standard solution of Hg(NO_3_)_2_ with EDTA solution using our proposed electrode as an indicator electrode at optimized conditions. The concentrations used are mentioned in the text.

The proposed Hg^2+^–SE based on BMPMP as an ionophore was employed as an indicator electrode in potentiometric titrations to test the electrode’s ability for monitoring mercury (II) concentration in aqueous solutions. Figure 11 shows potentiometric titration curve of 60 mL of Hg(NO_3_)_2_ (2.0 × 10*^–^*^3^ molL^–1^) with 3.0 × 10*^–^*^2^ molL^–1^of EDTA as a titrant. As seen in Figure 11, the potential response before the end point remains almost steady, due to the low concentration of EDTA in the solution. After the end point, the potential response remains constant which is referred to the low concentration of free Hg^2+^ ions in the solution. The end point of the titration was found to be ~4.0 mL. This indicates that the developed Hg^2+^–SE at optimized conditions is a suitable analytical tool for the potentiometric determination of Hg^2+^ ion in aqueous solutions.

### 3.10. Analytical Applications

The accuracy of Hg^2+^–SE designed in this work was tested using a dental amalgam capsule alloy (Dentsply Sirona company). Mercury concentration in this alloy estimated by the proposed Hg^2+^–SE was 41.8% (*w*/*w*) with a small difference from certified value (42.0%). However, student’s t-test showed that no significant difference between the two concentrations at the 95% confidence level because the tabulated value of t (2.78) is greater than the calculated one (2.65) for five replicate measurements. Therefore, electrode accuracy is acceptable from the point of view of analytical chemistry. Four real samples shown in Table 4 were used to evaluate the developed electrode. All samples were treated as above—mentioned and part of their aqueous solutions was subjected to potentiometric measurements using the developed Hg^2+^–SE as an indicator electrode. All samples were analyzed before and after spiking with known concentrations of Hg(II) ions. According to the results shown in Table 4, it is clear that the recovered amounts of the mercury from the real samples were almost quantified. Moreover, the samples were analyzed using ICP-OES. The results of ICP-OES measurements were in good agreement with those of Hg^2+^–SE as shown in Table 4. The statistical evaluation using F–test has been applied, and the results revealed that no statistical difference between two methods where the calculated values of F were always less than the tabulated F value (3.179) for ten replicate measurements. Therefore, we can say that the precision of both methods is statistically acceptable and there is no significant difference between them at the 95% confidence level.

### 3.11. Comparison with Previous Studies

The efficiency of many ISEs that use different ionophores was compared with analytical performance of electrode developed in this study. Comparison shown in Table 5 includes slope, detection limit, working pH, and selectivity. The developed Hg^2+^–SE offers much better features than the electrodes mentioned in the comparison. Moreover, the analytical performance of the proposed electrode in terms of sensitivity, recovery, and linear concentration range was compared with different analytical techniques [63,64,65,66,67,68,69]. The Hg^2+^–SE designed in the present study provides better performance than some of the methods mentioned in Table 6 without the need to use the extraction or preconcentration methodology. Moreover, the proposed electrode can operate at a wide range of pH values, therefore, it is suitable for analysis without the need to adjust sample pH in most cases. The good selectivity of developed Hg^2+^–SE may be attributed to the PVC membrane composition containing BMPMP as an ionophore. BMPMP molecule serves as a selective ligand for Hg^2+^ ions.

## 4. Conclusions

For the first time, Schiff base (BMPMP) is synthesized and used as a neutral carrier to design a new PVC membrane for Hg^2+^ ions. The interaction between BMPMP and Hg^2+^ ions in the PVC membrane matrix was studied by ATR–FTIR spectra, SEM images, and EDX spectra. The study of ATR–FTIR spectra recorded using the electrode membrane revealed that the Hg^2+^ ion could be coordinated with a BMPMP molecule through nitrogen and oxygen atoms. The analysis of SEM images and EDX spectra confirmed the presence of analyte in the membrane matrix. The membrane composition of 32% PVC, 64.5% *o*-NPOE, 2% BMPMP, and 1.5% NaTPB provides a better analytical performance with high selectivity towards Hg^2+^ ions over a wide concentrations range 9.33 × 10^–8^–3.98 × 10^–3^ molL^–1^ (0.0933–3980 µM). The electrode developed in this work offers a relatively fast response, less interference, reasonable long-term stability, and potential stability. The fabricated electrode was successfully applied for the determination of Hg(II) in real samples. 

## Figures and Tables

**Table 1:** Optimization of membrane composition of the proposed Hg^2+^-ISE based on the BMPMP ionophore.

ISE No.	Membrane Composition (%)				Slope (mV/Decade) ^a^	D.L. ^b^ (mol.L^−1^)	L.R. ^b^ (mol.L^−1^)	R.T. ^b^ (s)
	BMPMP	PVC	Plasticizer *o*-NPOE	Additive NaTPB				
1	0	32	68	0	0.61 ± 0.2	-	-	-
2	0	32	67	1	4.30 ± 0.2	-	-	-
3	1	32	67	0	17.07 ± 0.2	1.78 × 10^−6^	3.16 × 10^−6^–5.62 × 10^−4^	20
4	1	32	66	1	22.62 ± 0.2	3.16 × 10^−5^	4.47 × 10^−5^–3.16 × 10^−3^	10–15
5	1.5	32	66	0.5	25.92 ± 0.2	1.58 × 10^−6^	2.51 × 10^−6^–1.0 × 10^−3^	10–15
6	1.5	32	65	1.5	27.78 ± 0.2	1.58 × 10^−7^	6.31 × 10^−7^–7.08 × 10^−3^	>10
7	2	32	64.5	1.5	29.74 ± 0.2	3.98 × 10^−8^	9.33 × 10^−8^–3.98 × 10^−3^	<10
8	2	33	63.5	1.5	34.41 ± 0.2	1.58 × 10^−7^	2.82 × 10^−7^–1.00 × 10^−3^	10–15
9	2	34	62.5	1.5	32.62 ± 0.2	1.26 × 10^−5^	3.16 × 10^−5^–7.94 × 10^−3^	10–15

^a^ All slope values reported represent the mean and ±SD of three measurements. ^b^ D.L., L.R., and R.T. denote the lower detection limit, linear working range and response time respectively. The results are based on three replicate measurements.

**Table 2:** The influence of internal solution concentration on the potential of the optimized Hg^2+^–SE.

Concentration of Internal Solution (molL^–1^)	Slope (mV/Decade)	D.L. (molL^–1^)	L.R. (molL^–1^)	R.T. (s)
0.01	29.74 ± 0.2	3.98 × 10^−8^	9.33 × 10^−8^–3.98 × 10^−3^	5
0.001	27.16 ± 0.2	5.01 × 10^−5^	1.15 × 10^−4^–1.95 × 10^−2^	10
0.0001	17.63 ± 0.2	7.08 × 10^−6^	1.26 × 10^−5^–3.16 × 10^−3^	7

**Table 3:** Selectivity coefficients of proposed Hg^2+^–SE (ISE-7) for different interfering ions calculated using MPM at optimized conditions.

Interfering Ion, M	$K_{Hg,M}^{pot}$	Interfering Ion, M	$K_{Hg,M}^{pot}$
Hg^2+^	1.00	Fe^3+^	1.9 × 10^−4^
Na^+^	2.81 × 10^−5^	Ag^+^	1.5 × 10^−3^
K^+^	1.94 × 10^−5^	Cu^2+^	2.55 × 10^−4^
Ca^2+^	5.71 × 10^−5^	Zn^2+^	1.85 × 10^−5^
Mg^2+^	3.67 × 10^−6^	Co^2+^	7.67 × 10^−6^
Ba^2+^	1.86 × 10^−5^	Ni^2+^	1.98 × 10^−5^
Al^3+^	1.33 × 10^−5^	Fe^3+^	9.59 × 10^−5^
Cd^2+^	2.65 × 10^−5^	Cr^3+^	1.06 × 10^−6^
Mn^2+^	1.19 × 10^−5^	Pb^2+^	1.40 × 10^−5^

**Table 4:** The analysis of mercury in some real samples.

Sample *	Hg^2+^ Added (µM)	Hg^2+^ Ions Concentration (µM)			
		Hg^2+^–SE		ICP-OES	
		Found	Recovery (%)	Found	Recovery (%)
Tap water	0	ND	-	ND	-
	20	19.97 ± 0.15	99.85	20.03 ± 0.15	100.15
Red sea water	0	ND	-	0.040 ± 0.001	-
	26	25.77 ±0.25	99.11	26.1 ± 0.2	100.23
Compact fluorescent lamp (CFL)	0	1.89 ± 0.65	-	1.95 ± 0.18	-
	26	27.86 ± 0.36	99.88	28.1 ± 0.22	100.58

* Results are based on the average of four measurements ± standard deviation, ND: not detected.

**Table 5:** Comparison between some responses characteristics of the proposed Hg^2+^-SE based on BMPMP with the previously reported for Hg^2+^-SES based on various ionophores with the reported electrodes.

Ionophore	Slope (mV/decade)	L.R. (mol.L^−1^)	D.L. (mol.L^−^^1^)	Working pH	R.T. (s)	Significant Interference $\left(K_{Hg,M}^{pot}\right)$	Ref.
*meso*-tetramethyl-tetrakis-(4*N*,*N*-diethylacetamide) phenoxymethyl] calix[4]pyrrole	29.0	1.81 × 10^−7^–1.78 × 10^−3^	1 × 10^−^^5^	4.3–8.5	10	Ag^+^ (4.2 × 10^−^^2^)	[22]
calix[4]pyrrole amide derivative	29.10	6.15 × 10^−8^–2.28 × 10^−2^	6.0 × 10^−^^8^	3.2–8.8	5–8	-	[24]
1,3-diphenylthiourea	30.8	2.00 × 10^−6^–2.10 × 10^−4^	1.0 × 10^−^^6^	4.0	<30	Ag^+^ (3.1 × 10^−^^2^)	[25]
1-methyl-2-butylthioimidazolium bis(trifluoromethanesulphonyl)imide	6.70	1.00 × 10^−10–^1.00 × 10^−5^	4.1 × 10^−^^11^	2.0–3.0	5	-	[29]
1,2-bis-(*N’*-benzoylthioureido)cyclohexane	28.1	1.00 × 10^−5^–1.00 × 10^−1^	2.5 × 10^−^^6^	7.0	50–100	-	[32]
*O,O*-(2,2_-biphenylene) dithiophosphate alkyl	29.80	1.00 × 10^−9^–1.00 × 10^−1^	9.1 × 10^−^^10^	0.0–6.0	30	Fe^3+^ (1.2 × 10^−^^5^)	[33]
1-n-butyl-3-methylimidazolium tetrafluoroborate	29.3	5.00 × 10^−9^–1.00 × 10^−4^	2.5 × 10^−^^9^	2.0–4.3	~5	-	[35]
polypyrrole-PPy	23.7 ± 1.4	1.0 × 10^−9^–1.0 × 10^−2^	6.0 × 10^−^^10^	3.0	60	Ag^+^ (1.0 × 10^−^^3^)	[36]
bis [5-((4-nitrophenyl) azo salicylaldehyde)]	30 ± 1.0	7.00 × 10^−7^–5.00 × 10^−2^	7.0 × 10^−^^8^	1.0–3.5	<10	-	[42]
salicylaldehyde thiosemicarbazone	29	1.78 × 10^−6^–1.00 × 10^−1^	1.0 × 10^−^^6^	1.0–3.0	<30	Ag^+^ (3.1 × 10^−^^2^)	[43]
1-(2-hydroxy-1,2-diphenylethylidene)thiosemicarbazide	30.5	2.00 × 10^−6^–1.00 × 10^−2^	1.0 × 10^−^^6^	2.0–5.0	<30	Ag^+^ (2.7 × 10^−^^1^)	[44]
2-amino-6-purinethiol	29.4	7.00 × 10^−8^–1.00 × 10^−1^	4.4 × 10−8	2.0–4.5	10	Ag^+^ (1.5 × 10^−^^2^); Cu^2+^ (3.1 × 10^−^^2^); Pb^2+^ (5.0 × 10^−^^2^)	[45]
bis-salicyladehydediaminjodipropylamine	30.5 ± 0.4	9.50 × 10^−7^–6.40 × 10^−2^	7.0 × 10^−^^7^	1.5–3.5	10–25	Ag^+^ (3.1 × 10^−^^2^)	[46]
poly-*o*-toluidine into zirconium phosphoborate	28.0	1.0 × 10^−7^–1.0 × 10^−1^	7.08 × 10^−^^8^	2.5–10.0	6	K^+^ (6.4 × 10^−^^2^)	[51]
1-undecanethiol assembled Au substrate	28.83	1.00 × 10^−8^–1.00 × 10^−4^	4.5 × 10^−^^9^	6.0	~30	-	[54]
trioctylmethylammonium thiosalicylate	44.08	1.00 × 10^−9^–1.00 × 10^−2^	1.0 × 10^−^^10^	3.0–9.0	~5	Ag^+^ (8.9 × 10^−^^4^); Cu^2+^ (8.9 × 10^−^^4^); Pb^2+^ (9.6 × 10^−^^4^)	[60]
dithio derivatized macrotricyclic	29.4 ± 0.4	1.00 × 10^−6^–1.00 × 10^−1^	4.0 × 10^−^^7^	1.5–4.0	<10	Ag^+^ (1.3 × 10^−^^2^)	[70]
poly-o-anisidine Sn(IV) tungstate	21.0	1.00 × 10^−7^–1.00 × 10^−1^	1.00 × 10^−7^	4.0–8.0	30	_____	[71]
4-Bromo-2-[(4-methoxyphenylimino)methyl]phenol	29.74	9.33 × 10^−8^–3.98 × 10^−3^	3.98 × 10^−8^	3.0–7.0	<10	_____	P.W.

**Table 6:** Comparisons of analytical results for the detection of Hg^2+^ by using other methods.

Materials	Method	Sample	L.R. (molL^−1^)	D.L. (mgL^−^^1^)	pH	Recovery	Ref.
AuNPs, DNA	spICP-MS	tap water	1.0 × 10^−7^–1.0 × 10^−6^	3.1 × 10^−^^8^	-	110 ± 10	[63]
T-gCNQDs	Fluorescence	pond water	1.0 × 10^−8^–5.0 × 10^−7^	1.5 × 10^−10^	7.2	95	[64]
pyrrolidinedithiocarbamate (PDC)	D-DLLME/GFAAS	rice	0.01–0.3	1.8 × 10^−7^	6.0	98	[65]
AuNPs, APTES	Colorimetric	river water	0–9.23 × 10^−8^	1.0 × 10^−8^	7.2	96.4	[66]
-	PVG-CVAAS	DORM-1	1.75 × 10^−7^–1.69 × 10^−6^	1.4 × 10^−9^	-	92.7	[67]
-	CKM	wastewater	1.0–30.0 × 10^−6^	1.5 × 10^−7^	4	97.93	[68]
graphene nanosheets	DMSPE	Fresh water	1.9 × 10^−9^–5.19 × 10^−6^	1.9 × 10^−9^	-	95.0<	[69]

spICP-MS: single-particle inductively-coupled plasma/mass spectrometry; D-DLLME/GFAAS: displacement-dispersive liquid-liquid microextraction-graphite furnace atomic absorption spectrometry; PVG-CVAAS: Photochemical Vapor Generation-Cold Vapor Atomic Absorption Spectrometry; CKM: catalytic kinetic method; DMSPE: Dispersive Micro-Solid Phase Extraction.

## Data Availability

Not Applicable.

## Supplementary Materials

The following are available online at https://www.mdpi.com/article/10.3390/s21093020/s1.
