# Supplementary material for: Design and Characterization of Electrochemical Sensor for the Determination of Mercury(II) Ion in Real Samples Based upon a New Schiff Base Derivative as an Ionophore
Source: Sensors (Basel). 2021 Apr 25;21(9):3020. doi: 10.3390/s21093020 (PMC8123339; doi:10.3390/s21093020)
Supplement: Supplementary file 1 [file sensors-21-03020-s001.zip › sensors-1176902-supplementary.pdf]

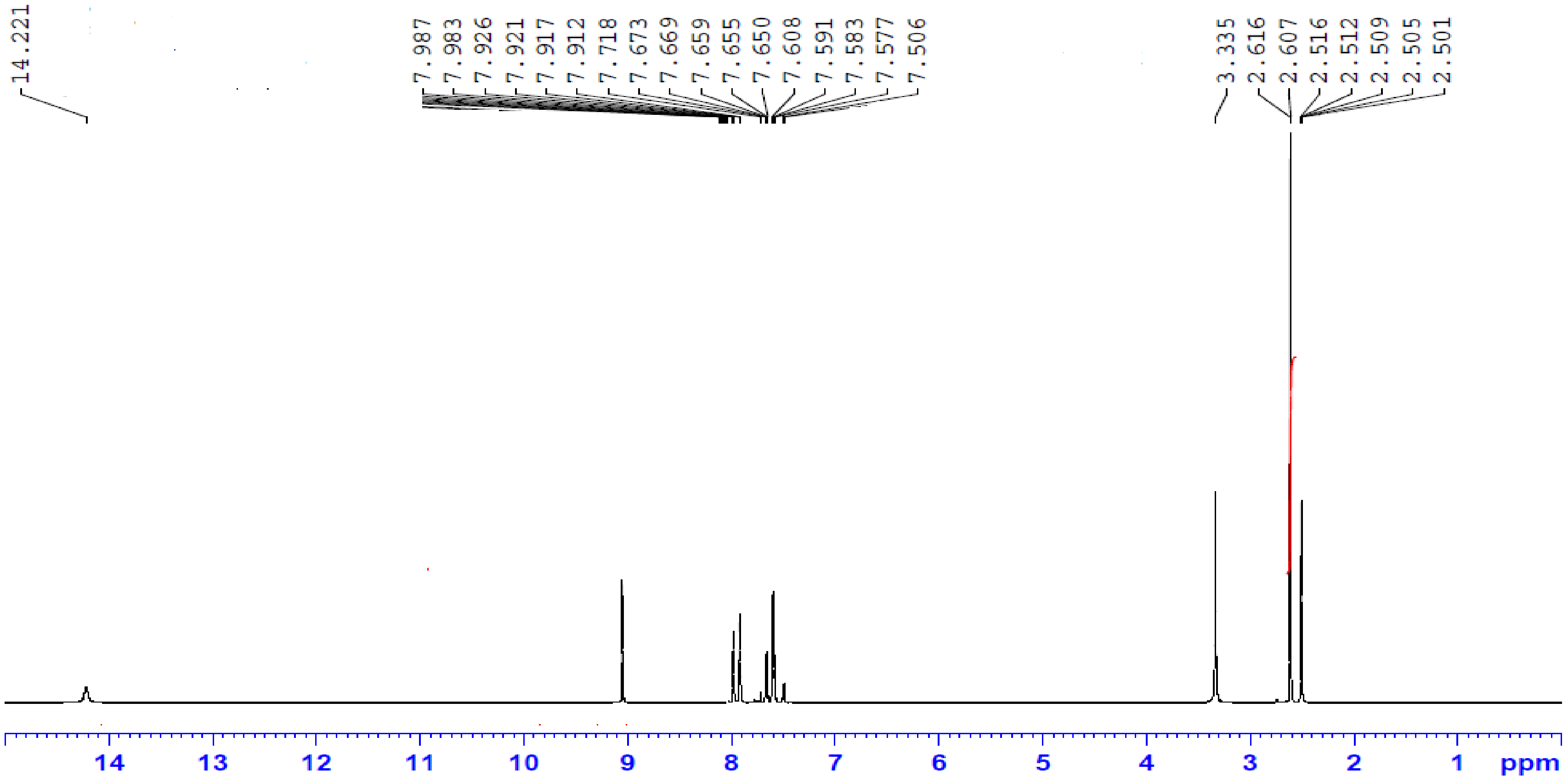

^{1}H-NMR spectrum of BMPMP in DMSO-d_6 solvent

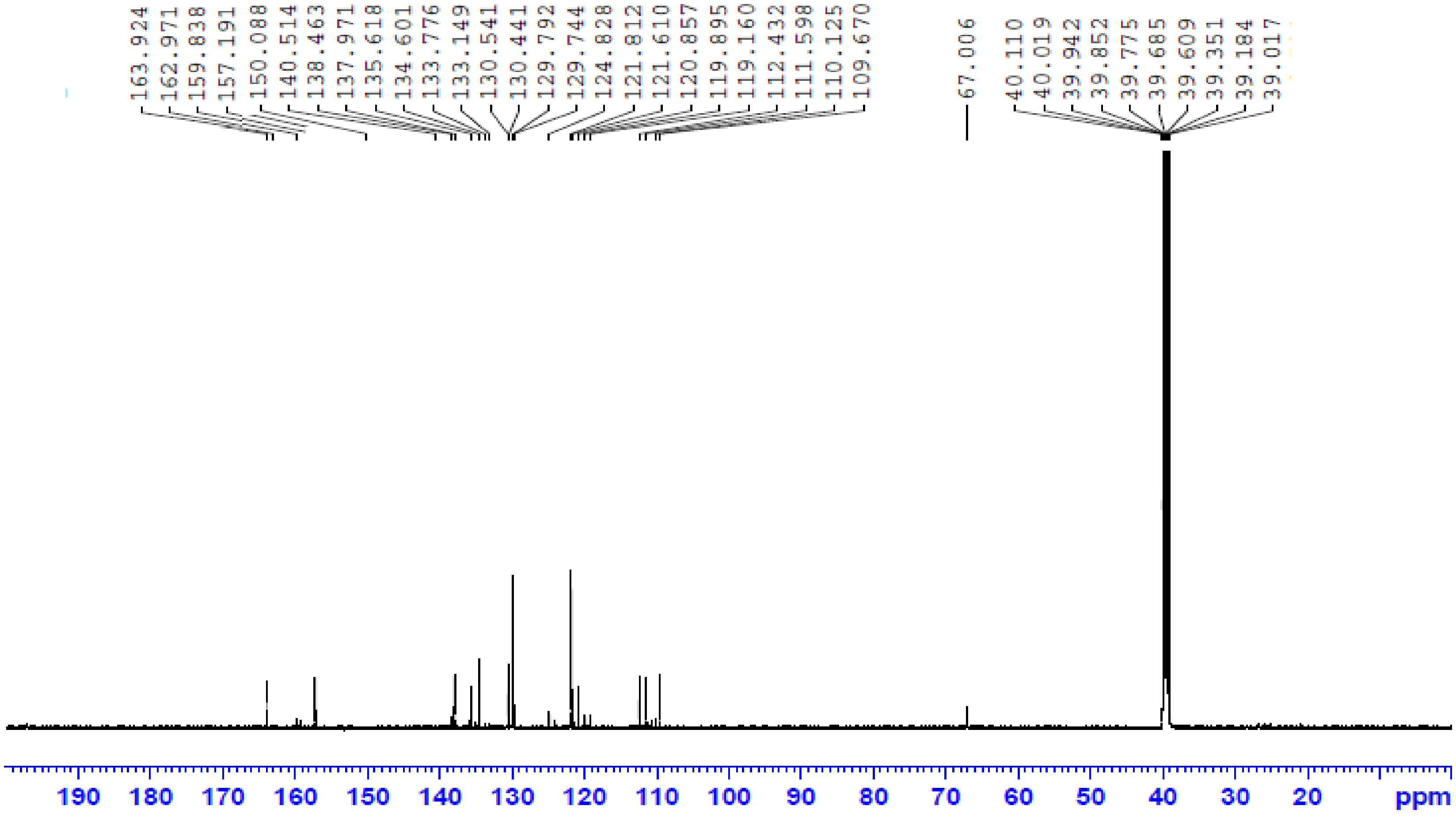

^{13}C-NMR spectrum of BMPMP in DMSO-d_6 solvent
